# Ginsenosides for the Management of Metabolic Dysfunction-Associated Fatty Liver Disease: A Research Update

**DOI:** 10.3390/nu18111806

**Published:** 2026-06-03

**Authors:** Ke Zhang, Zenghui Qin, Qingjun Guo, Jiazhi Lu, Huiyu Luo, Longying Zha

**Affiliations:** Department of Nutrition and Food Hygiene, National Medical Products Administration (NMPA) Key Laboratory for Safety Evaluation of Cosmetics, Guangdong Provincial Key Laboratory of Tropical Disease Research, School of Public Health, Southern Medical University, 1023–1026 Shatai South Road, Guangzhou 510515, China; kokozhang@126.com (K.Z.); 18174731970@163.com (Z.Q.); guoqingjun2000@163.com (Q.G.); yingzui0333366@163.com (J.L.); luohuiyu0414smu@163.com (H.L.)

**Keywords:** ginsenosides, metabolic dysfunction-associated fatty liver disease (MAFLD), oxidative stress, inflammation, gut–liver axis

## Abstract

**Background**: Metabolic-associated fatty liver disease (MAFLD) has a high prevalence of 30–40% in China and Asia, with a complex pathogenesis and no specific therapeutic drugs. Phytochemicals have become a research hotspot for MAFLD prevention, and ginsenosides, the core active components of Panax ginseng, show great potential in anti-MAFLD research. This review aims to comprehensively clarify the key mechanisms and targets of ginsenosides in preventing and treating MAFLD, to provide a theoretical basis for their application in metabolic diseases, and to promote the development of natural phytochemical resources. **Method**: The literature review method was adopted to sort out the regulatory effects and molecular targets of ginsenosides in multiple pathological processes of MAFLD from published studies. **Results**: Ginsenosides regulated MAFLD through multi-pathway and multi-target effects: antioxidant regulation occurred via Nuclear factor E2-related factor 2 (Nrf2)/Silent information regulator 1/6 (SIRT1/6) pathways, and anti-inflammatory regulation was achieved by inhibiting the Nuclear factor kappa-B (NF-κB)/NOD-like receptor family pyrin domain containing 3 (NLRP3) inflammasome. Additionally, the measures adopted improved insulin resistance and lipid metabolism disorder, suppressed hepatocyte apoptosis/pyroptosis, repaired autophagy, alleviated hepatocyte senescence, and reshaped gut microbiota to restore gut–liver axis homeostasis. **Conclusions**: Ginsenosides have good potential for MAFLD prevention and treatment, but there is a prominent lack of human clinical evidence as most existing studies are only based on in vitro cell and in vivo animal models, and the synergistic mechanisms among different ginsenoside components remain unclear. Future research needs multi-omics analysis, formulation optimization, and large-sample clinical trials, and ginsenosides have broad application prospects in MAFLD intervention.

## 1. Introduction

Metabolic dysfunction-associated fatty liver disease (MAFLD), formerly known as non-alcoholic fatty liver disease (NAFLD), was proposed to be renamed in 2020. It is defined as hepatic steatosis accompanied by overweight/obesity, type 2 diabetes mellitus (T2DM), or metabolic dysfunction. The new nomenclature can more precisely define the MAFLD patient population and facilitate disease classification for this patient group. Compared with the old name, MAFLD no longer has exclusivity but rather highlights the critical role of metabolic factors in the occurrence and progression of the disease [1]. A 2022 study indicated that the overall prevalence of MAFLD in China is approximately 35.58% [2], and other studies have reported that its incidence in Asian populations ranges from 30% to 40% [3].

The pathogenesis of MAFLD is extremely complex. The latest prevention and treatment guidelines suggest that modifying dietary patterns and lifestyles is the primary measure for MAFLD intervention. MAFLD is associated with numerous chronic diseases. Currently, drugs mainly focus on symptomatic treatment, and there are no specific medications available. Therefore, researching the prevention and treatment of MAFLD from a nutritional perspective holds significant importance [4].

Phytochemicals derived from daily diets and traditional Chinese medicines are secondary metabolites produced during plant metabolism. They no longer directly participate in plant metabolic processes but exhibit significant biological activities. In recent years, some phytochemicals (such as polyphenols and saponins) have been reported to possess biological activities, including hepatoprotection, anti-inflammation, antioxidation, and lipid-lowering effects, which can ameliorate MAFLD, making them a current hotspot in nutritional research [5,6,7].

Ginseng is a high-value precious medicinal herb—a perennial plant of the Panax genus in the Araliaceae family—and has been hailed as the “King of Herbs” since ancient times. It is listed as a top-grade medicine in *Shennong Ben Cao Jing*, with the effects of “tonifying the five zang-organs, calming the spirit, stabilizing the soul, relieving palpitations, dispelling pathogenic qi, improving eyesight and intelligence, and prolonging life with long-term use” [8]. Modern research has confirmed that various ginsenosides abundant in its rhizomes are the core pharmacodynamic substances responsible for ginseng’s efficacy; these are the active components studied most in-depth [9]. Panax ginseng (Asian or Korean ginseng), Panax quinquefolius (America ginseng), and Panax notoginseng (Sanchi ginseng) are the three best-selling medicinal plants rich in ginsenosides. In addition to the abovementioned species, Panax japonicus, Panax vietnamensis, and Panax zingiberensis are also used [10]. These species belong to the Panax genus of Araliaceae.

A prospective randomized clinical trial assessed the anti-inflammatory and anti-fatigue properties of Korean Red Ginseng (KRG) in subjects with non-alcoholic fatty liver disease (NAFLD). KRG treatment led to marked reductions in key hepatic biochemical indicators, including aspartate aminotransferase (AST), alanine aminotransferase (ALT), and gamma-glutamyl transferase (γ-GT). Improvements in ALT and γ-GT levels were particularly pronounced in KRG recipients with a body mass index (BMI) of 25 kg/m^2^ or above. Moreover, KRG administration lowered serum tumor necrosis factor-alpha (TNF-α) concentrations and raised serum adiponectin levels in NAFLD patients [11]. As adiponectin is a well-established biomarker of metabolic syndrome, the increased adiponectin levels in the KRG group support the therapeutic potential of KRG for fatty liver disease.

As a time-honored medicinal plant, Panax ginseng has an extensive history in the development and application of health-promoting products. At present, a wide variety of products utilizing ginseng or ginseng extract as the core raw material are available on the market, covering foods, pharmaceuticals, cosmetics, and other categories. Market research data indicate that, driven by the continuous rise in consumers’ awareness of health and wellness, the global ginseng product market has maintained a steady annual growth rate of 5–7% in recent years. In the food sector, ginseng features high adaptability and diverse application forms, and can be incorporated into various dietary preparations. It is often used as a distinctive ingredient in soups and stews in high-end catering, enhancing flavor while endowing products with health benefits. In the pharmaceutical field, the research and development of ginseng-based drugs is progressing steadily, and clinical trials have been conducted in multiple countries to explore its therapeutic potential for chronic diseases such as diabetes and cardiovascular diseases. In addition, more than 80% of the total ginseng production in South Korea is processed into food products, such as red ginseng, dried ginseng, and ginseng extract, ginseng cream, ginseng powder, ginseng candies, ginseng herbal products, ginseng beverages, and ginseng wine [12].

**Figure 1:**
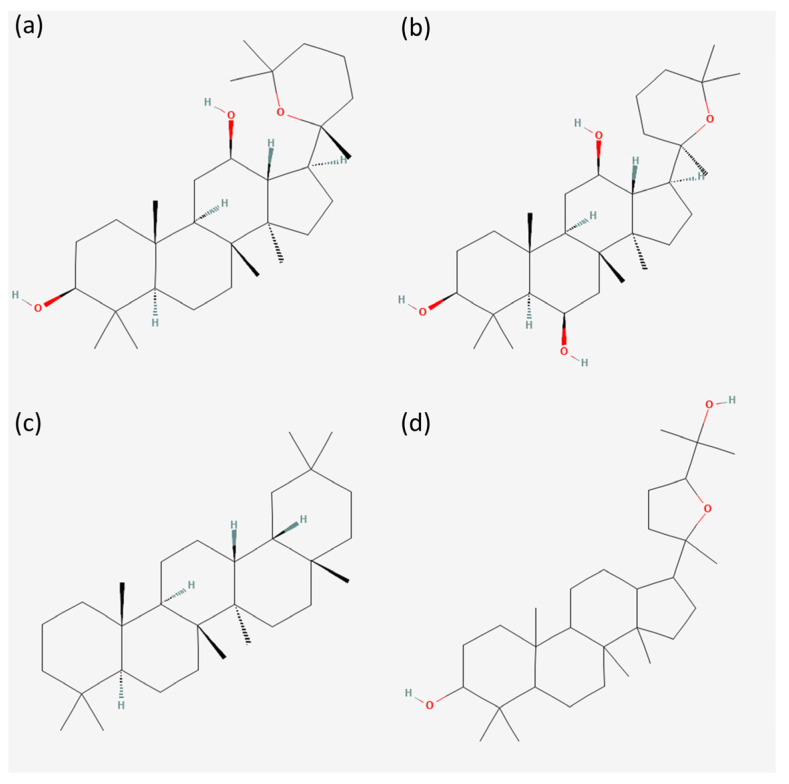
Chemical Structures of ginsenoside skeleton. (**a**) Protopanaxadiol (PPD); (**b**) Protopanaxatriol (PPT); (**c**) Ocotillol type; (**d**) Oleanolic acid type.

Ginsenosides are the major bioactive components of Panax ginseng and serve as the core indicators for evaluating its quality and medicinal and nutritional values. The root is the traditional medicinal part of Panax ginseng, while in recent years, several novel ginsenosides have also been identified in the above-ground parts of the plant (e.g., stems, leaves, flowers and flower buds). Ginsenosides belong to triterpenoid saponins, which share a similar basic structure and all contain a sterane steroid nucleus. Based on the structural differences in their aglycones, they are classified into three categories: dammarane type, oleanolic acid type, and ocotillol type. Dammarane-type ginsenosides can be further subdivided into protopanaxdiol (PPD) and protopanaxatriol (PPT) subtypes according to the different positions of substituents attached to the aglycone. For instance, PPD-type ginsenosides include Rb1, Rb2, Rb3, Rc, Rd, Rh2, R3 and F2, whereas PPT-type ginsenosides comprise Re, Rf, Rg1, Rg2, Rh1 and F1 [13]. Among these, Rb1, Rb2, Rc, Rd, Re and Rg1 account for more than 90% of the total ginsenoside content, and Rb1, Rd, Re, Rg1, R3, Rh1 and Rh2 have been the most frequently investigated in relevant research [14] (Figure 1).

Toxicity data specifically targeting individual ginsenosides remain scarce. However, ginseng is widely recognized for its low systemic toxicity and is considered suitable for use as both a daily nutritional supplement and a clinical therapeutic agent. In a 4-week repeated-dose toxicity study in rats, Korean red ginseng extract (rich in ginsenosides) was administered at 0, 500, 1000, and 2000 mg/kg/day. No mortality was observed in either male or female rats, even at the highest dose [15]. Collectively, ginsenosides may possess high biological activity and low organ toxicity and can be safely used as a therapeutic agent for liver diseases.

Ginsenosides show consistent pharmacokinetic characteristics in vivo. Oral absorption is poor with low absolute bioavailability, caused by large molecular mass, high hydrogen-bonding capacity, low intestinal permeability, weak water solubility, and rapid degradation or biotransformation by gastric acid and intestinal flora [16,17,18]. They distribute rapidly into tissues including heart, liver, lung, and kidney, and some can cross the blood–brain barrier [19]. Intestinal microflora-mediated deglycosylation is the core metabolic pathway, converting major ginsenosides into active minor ones [20]. Excretion routes differ: Rb2 and Rd are excreted mainly via urine, while Rg1 is eliminated primarily through bile [21,22]. Elimination rates vary [23]. These absorption/distribution/metabolism/excretion (ADME) traits restrict their oral bioavailability and clinical application.

Compared with previously published reviews focusing on ginsenosides intervening MAFLD pathogenesis, this narrative review presents distinct scientific innovations and targeted improvements, overcoming prominent limitations of existing relevant reviews. The unique scientific contributions of this review are summarized as follows. First of all, different from previous reviews that separately list dispersed signaling pathways or individual ginsenoside monomers, this review reconstructs the overall regulatory network strictly following the classical multiple-hit pathogenesis of MAFLD. It systematically connects initial metabolic disturbance, secondary oxidative and inflammatory injury, terminal hepatocyte fate disorder, and persistent gut–liver axis dysfunction in a progressive pathological cascade, establishing an integrated holistic regulatory framework rather than isolated mechanical descriptions, which conforms to the actual progressive pathological characteristics of MAFLD. Secondly, unlike most existing reviews that only summarize positive preclinical findings without objective literature evaluation, this review adds hierarchical critical assessment throughout all chapters. It comprehensively analyzes inconsistent experimental results, inherent limitations of cell and animal models, publication bias of existing preclinical evidence, as well as dose gaps between preclinical pharmacological concentration and human practical administration, transforming a simple literature compilation into a critical and integrated review of current research evidence. Thirdly, this review integrates gut microbiota-mediated ginsenoside biotransformation into the whole regulatory network of MAFLD for targeted discussion, and further proposes staged, forward-looking translational research directions combined with clinical medication norms and pharmacopoeia administration standards. It clarifies the practical application positioning of ginsenosides as early preventive and adjuvant intervention agents instead of specialized therapeutic drugs, making up for the lack of targeted translational prospects in previous reviews. In general, this review provides a more systematic, critical, and clinically referential summary for subsequent basic research and translational exploration of ginsenosides against MAFLD.

## 2. Literature Survey

A thorough search of the scientific literature was conducted in PubMed, Web of Science, CNKI, and Wanfang Data using various combinations of the following keywords: “Ginseng”, “Panax”, “saponin”, “Ginsenoside”, “Non-alcoholic fatty liver disease”, “Non-alcoholic steatohepatitis”, “Metabolic dysfunction-associated fatty liver disease”, “Metabolic dysfunction-associated steatotic liver disease”, “Metabolic Associated Steatohepatitis”, “Metabolic Diseases”, and “Gut microbiota”. The studies selected were based on their relevance to the topics covered in this review. Articles published in English and Chinese between January 2000 and January 2026 were included, with a special focus on those offering insights into dietary Ginsenosides, including their diversity, mechanism, and potential hepatoprotective effects. This review is a narrative review aimed at integrating the main research findings, mechanisms, and future directions in the field, rather than a systematic evaluation. Therefore, we did not adopt the complete retrieval strategy or formal bias risk assessment tool required for a systematic review.

## 3. Ginsenosides Target the Initial “First Hit”: Insulin Resistance and Lipid Metabolism Disorders in MAFLD

As the initiating event in MAFLD pathogenesis, insulin resistance and dysregulated lipid metabolism represent the first critical “hit” that triggers subsequent pathological cascades. By targeting these early-stage metabolic abnormalities, ginsenosides hold the potential to halt disease progression at its origin.

### 3.1. Improvement of Insulin Resistance

As the core initial hit in the multiple-hit hypothesis of MAFLD, insulin resistance (IR) constitutes the central pathological basis shared by MAFLD and other metabolic diseases and acts as the pivotal link connecting hepatic steatosis with systemic metabolic disorders. Based on current research, ginsenosides can effectively alleviate MAFLD-related IR by multi-target activation of the insulin signaling pathway, the regulation of key glucose metabolism molecules, and improvement of the metabolic microenvironment.

#### 3.1.1. Activation of the Insulin Signaling Pathway

The primary mechanisms by which ginsenosides regulate insulin resistance are activating core molecules of the insulin signaling pathway and restoring insulin sensitivity. Li et al. confirmed in the HFD-induced MAFLD mouse model that ginsenoside Rb1 can significantly upregulate the expression and secretion level of adiponectin in adipose tissue [24]. As an insulin sensitivity regulator, adiponectin can activate liver Adenosine monophosphate-activated protein kinase (AMPK) phosphorylation, enhance Insulin receptor substrate-1 (IRS-1) tyrosine phosphorylation (inhibit serine phosphorylation), and restore Phosphatidylinositol 3-kinase (PI3K)/Protein kinase B (Akt) pathway activity. Moreover, adiponectin-neutralizing antibodies can significantly weaken the inhibitory effect of Rb1 on hepatocyte triglycerides (TG) accumulation, and the IR improvement effect of Rb1 is completely lost in adiponectin knockout mice, clarifying that adiponectin is a key molecule for Rb1 to alleviate IR [24]. Similarly, in HFD-induced MAFLD mice, Rg2 intervention can significantly reduce fasting blood glucose and insulin levels, and improve the results of glucose tolerance test (GTT) and insulin tolerance test (ITT). Its effect is completely eliminated in liver-specific Silent information regulator 1 (SIRT1) knockout mice, confirming that SIRT1 is a key target for Rg2 to regulate IR [25].

Notably, all the above findings are derived from conventional rodent models. Differences in species and physiological characteristics between experimental animals and humans may limit the extrapolation of these molecular mechanisms. In addition, current studies mainly focus on single ginsenoside monomers, and the combined effects of multiple components remain poorly understood.

#### 3.1.2. Regulation of Gluconeogenesis and Glucose Utilization

In addition to activating core molecules of the insulin signaling pathway, ginsenosides can also reduce hepatic gluconeogenesis and promote glucose utilization by regulating key glucose-metabolizing enzymes and transcription factors. The excessive activation of hepatic gluconeogenesis is an important cause of hyperglycemia in the IR state, which is mainly regulated by key enzymes such as phosphoenolpyruvate carboxykinase (PEPCK) and glucose-6-phosphatase (G6Pase). In fructose-induced MAFLD mice, ginsenoside C-K can downregulate the mRNA and protein expression of hepatic PEPCK and G6Pase by activating the Liver kinase B1 (LKB1)/AMPK pathway, and promote Glucose transporter 4 (GLUT4) membrane localization to enhance hepatocyte glucose uptake [26]. A study by Shi found that ginsenoside Rg5 can promote hepatic energy metabolism by activating the LKB1/AMPK/mammalian target of rapamycin (mTOR) signaling pathway. In HFD-induced MAFLD mice, Rg5 can reduce fasting blood glucose and HOMA-IR index in a dose-dependent manner and improve glucose intolerance. Fecal microbiota transplantation (FMT) experiments confirmed that the gut microbiota regulated by Rg5 (such as increased abundance of Bacteroide and Akkermansia) can confer its IR improvement effect, suggesting that gut microbiota is an important mediating link in Rg5-mediated alleviation of IR [27].

Existing evidence indicates that ginsenosides regulate glucose metabolism via the LKB1/AMPK axis across different models, yet the effective doses vary markedly among studies. Meanwhile, gut microbiota acts as an important mediator in this process, and individual differences in intestinal flora may cause inconsistent drug responses in practical applications.

#### 3.1.3. Reduction in Lipotoxicity and Oxidative Damage

Furthermore, ginsenosides can alleviate the induction of IR by lipotoxicity and oxidative damage by improving hepatic lipid metabolism disorders and oxidative stress. Lipotoxicity, caused by the accumulation of FFA and TG in the liver, is one of the core inducers of IR, and oxidative stress can further damage the insulin signaling pathway [28]. Ginsenoside Rd upregulates peroxisome proliferator-activated receptor α(PPAR-α)-mediated fatty acid oxidation and inhibits the expression of 3-hydroxy-3-methylglutaryl-CoA reductase (HMGCR), a key enzyme in hepatic cholesterol synthesis, by activating Silent information regulator 6 (SIRT6) deacetylase activity, reducing hepatic TG and FFA contents in HFD-induced MAFLD mice, and improving insulin-mediated glycogen synthesis. SIRT6 knockout can weaken its IR improvement effect [28].

This regulatory pathway has been validated in HFD-fed mice, but relevant research across multiple animal strains and cell lines is still insufficient. Whether lipotoxicity and oxidative stress serve as universal upstream drivers of insulin resistance under different pathological backgrounds needs further verification.

The above studies have confirmed that ginsenosides can effectively alleviate MAFLD-related insulin resistance through interventions such as activating the insulin signaling pathway, regulating key glucose metabolism molecules, and improving lipid metabolism disorders. The effects of some saponins (such as Rb1 and Rg2) are mediated by key molecules such as adiponectin and SIRT1, providing clear experimental evidence for the targeted treatment of MAFLD complicated with IR. Nevertheless, current preclinical evidence still has obvious limitations. Most relevant animal experiments adopt short-term intervention cycles and lack long-term efficacy and safety monitoring; all conclusions are derived from cell and rodent models without human sample verification. In addition, existing studies mainly focus on single ginsenoside monomers, while the synergistic or antagonistic effects among different components remain poorly clarified, hindering further clinical formulation development.

Collectively, preclinical studies have fully demonstrated the insulin-sensitizing effects of ginsenosides. However, the overall evidence still has limitations: most trials adopt short-term intervention and lack long-term efficacy and safety observation; relevant human data is completely absent, and it remains unclear whether the targets and pathways identified in preclinical models can be replicated in MAFLD patients.

### 3.2. Regulation of Lipid Metabolism Disorders

Hepatic lipid metabolism disorder, the direct initial hit in the multiple-hit hypothesis of MAFLD, is the direct cause of hepatocyte lipotoxicity and hepatic steatosis, and constitutes one of the core pathological features of MAFLD. It is mainly manifested as the abnormal accumulation of TG and free fatty acids (FFA) in hepatocytes. The underlying mechanisms involve enhanced hepatic lipid synthesis, attenuated fatty acid oxidation, and impaired lipid transport. These events interact to form a vicious cycle of “lipid accumulation–metabolic disorder”, thereby accelerating disease progression.

#### 3.2.1. Inhibition of Lipid Synthesis

In terms of inhibiting hepatic lipid synthesis, ginsenosides mainly exert their effects by downregulating the expression of sterol regulatory element-binding protein 1c (SREBP1c) and its target genes. SREBP1c is the core transcription factor regulating fat synthesis, which can activate the expression of key enzymes such as fatty acid synthase (FAS) and acetyl-CoA carboxylase (ACC) to promote TG synthesis [29]. In mice with MAFLD induced by a Western diet, ginsenoside Rk1 downregulates the mRNA and protein expression of SREBP1c, FAS, and ACC by targeting fatty acid translocase fatty Acid translocase CD36 (CD36), and restores AMPK phosphorylation, significantly reducing hepatic TG content [29]. Ginsenoside C-K downregulates the expression of SREBP1c and FAS in fructose-induced MAFLD mice by activating the LKB1/AMPK pathway [26]. In type 2 diabetic OLETF rats (complicated with MAFLD), C-K intervention can significantly reduce hepatic TG and total cholesterol (TC) levels, an effect that can be reversed by the AMPK inhibitor Compound C [30]. Ginsenoside Rg5 can also reduce hepatic lipid synthesis by downregulating SREBP1c expression in HFD-induced MAFLD mice through activating the LKB1/AMPK/mTOR signaling pathway [27].

Current studies consistently confirm that ginsenosides suppress lipid synthesis via the SREBP1c pathway, but discrepancies exist in the effective concentrations and intervention cycles across different diet-induced models. The off-target effects of related pathways also lack systematic evaluation.

#### 3.2.2. Promotion of Fatty Acid β-Oxidation

In terms of promoting fatty acid β-oxidation, ginsenosides are mainly achieved by activating PPAR-α and its downstream target genes [31]. In FFA-induced HepG2 cells, ginsenoside Rg1 can reduce intracellular TG content [32]. In oleic acid, combined with high glucose-induced HepG2 cells, ginsenoside Rb2 can restore autophagic flux and promote lipid droplet degradation and fatty acid oxidation [33]. In HFD-induced MAFLD mice, ginsenoside Rg1 promotes fatty acid oxidation by upregulating the mRNA and protein expression of PPAR-α, carnitine palmitoyltransferase 1 (CPT1), and acyl-CoA oxidase 1 (ACOX1) [34]. In MAFLD rats, Rg1 can significantly increase hepatic CPT1 and ACOX1 activities, improving fatty acid oxidation function [32]. Ginsenoside Rb2 upregulates the expression of PPAR-α and CPT1 in db/db mice (complicated with MAFLD) by inducing SIRT1 and activating AMPK [35]. In HFD-induced MAFLD mice, Rb2 can also upregulate the expression of cholesterol 7α-hydroxylase (CYP7A1), promote the conversion of cholesterol to bile acids, and reduce hepatic cholesterol accumulation [36].

Data from in vitro cell models and in vivo animal models jointly support the pro-oxidation effect of ginsenosides. Nevertheless, most in vitro experiments use high-dose drug intervention, a method that struggles to match the actual exposure level of ginsenosides in human daily intake.

#### 3.2.3. Enhancement of Lipid Transport

In terms of enhancing lipid transport, ginsenosides can promote hepatic lipid efflux by upregulating the expression of apolipoproteins and lipid transport proteins. Apolipoprotein B100 (ApoB100) is the core component of very low-density lipoprotein (VLDL), which can mediate the transport of hepatic TG to the blood; microsomal triglyceride transfer protein (MTTP) is a key protein for VLDL assembly. In FFA-induced HepG2 cells, ginsenoside Rg1 can upregulate the mRNA and protein expression of ApoB100 and MTTP to enhance lipid transport [34]. Ginsenoside extract (GE) improves lipid metabolism in HFD-induced MAFLD mice by upregulating the expression of hepatic CPT-1a (fatty acid oxidation gene) and ApoB100 and inhibiting SREBP1c [37]. The saponin fraction (SF) from the aerial parts of Japanese ginseng promotes lipid transport and reduces hepatic TG content in HFD-induced rat MAFLD models by activating the AMPK/ACC pathway and upregulating MTTP expression [38].

Existing research covers ginsenoside monomers, total extracts, and saponin fractions, but direct comparative studies among different components are scarce. It is difficult to clarify which component plays a dominant role in regulating lipid transport.

In summary, ginsenosides can improve MAFLD lipid metabolism disorders through multi-dimensional interventions based on “inhibiting lipid synthesis–promoting fatty acid oxidation–enhancing lipid transport”. Different saponins (Rk1, CK, Rg1, Rb2, etc.) exert synergistic effects through different specific targets in preclinical models, providing abundant evidence for the regulation of lipid metabolism disorders in MAFLD. However, these preclinical findings face prominent translational bottlenecks. In vitro cellular experiments commonly use excessively high drug concentrations that cannot match actual human oral exposure doses, and diverse animal models lead to inconsistent effective doses of identical ginsenosides. Moreover, existing research rarely reports negative or conflicting experimental data, causing potential publication bias and reducing the overall reliability of current evidence.

As the initial hit of MAFLD, insulin resistance and lipid metabolic disorders are the main research directions of current ginsenoside studies. On the whole, relevant preclinical evidence is dominated by positive results, while negative or inconsistent data are rarely reported. The single experimental model, relatively homogeneous research design, and the lack of human clinical trials collectively restrict the clinical interpretability of existing conclusions. Follow-up research should focus on unifying experimental conditions, exploring component compatibility, and carrying out dose conversion research to lay a foundation for translating preclinical findings into practical intervention strategies for early MAFLD.

## 4. Ginsenosides Block Secondary “Second Hit”: Oxidative Stress and Inflammatory Responses in MAFLD

Building on initial metabolic stress, oxidative stress and chronic inflammation constitute the secondary “hit” that amplifies hepatic injury and drives progression from simple steatosis to NASH. Ginsenosides exert multi-level inhibitory effects on these interconnected pathological processes.

### 4.1. Regulation of Oxidative Stress

As the key secondary hit in the multiple-hit hypothesis of MAFLD, oxidative stress serves as the critical bridge linking metabolic disorders to hepatic inflammation and cellular injury, and acts as a core driver in the occurrence and development of MAFLD. The accumulation of free fatty acids (FFA) leads to overload of β/ω-oxidation in mitochondria, peroxisomes, and endoplasmic reticulum, resulting in reactive oxygen species (ROS) burst [39]. Meanwhile, the antioxidant system is depleted, causing lipid peroxidation, DNA and membrane structure damage, and further inhibiting mitochondrial function [40]. Excessive ROS not only directly induce lipid peroxidation (e.g., generating malondialdehyde (MDA)), oxidatively modify proteins and DNA, and damage hepatocyte structure and function, but also trigger inflammatory responses and lipid metabolism disorders, forming a vicious cycle that promotes the progression of the disease from simple steatosis to metabolic dysfunction-associated steatohepatitis (MASH) and liver fibrosis [41].

#### 4.1.1. Activation of the Nrf2-ARE Signaling Pathway

The regulatory effect of ginsenosides on oxidative stress in MAFLD is first reflected in the activation of the nuclear factor E2-related factor 2 (Nrf2)-antioxidant response element (ARE) signaling pathway, which plays a key role in ginsenoside-regulated oxidative stress responses [42]. Nrf2 is the core transcription factor regulating antioxidant stress in the body. Under physiological conditions, it binds to Kelch-like ECH-associated protein 1 (Keap1) in the cytoplasm and remains dormant. When stimulated by oxidative stress signals such as ROS, Nrf2 dissociates from Keap1 and translocates into the nucleus, binds to the ARE sequence, and initiates the expression of downstream antioxidant enzymes and detoxification proteins such as superoxide dismutase (SOD), glutathione peroxidase (GSH-Px), catalase (CAT), and heme oxygenase-1 (HO-1), thereby scavenging excessive ROS and reducing oxidative damage [42].

A study by Gao et al. found that in the FFA-induced HepG2 cell MAFLD model, ginsenoside Rg1 can reduce intracellular MDA content (a marker of lipid peroxidation) and significantly increase SOD activity [43].

In the high-fat-diet (HFD)-induced MAFLD mouse model, Rg1 intervention can also significantly upregulate the mRNA and protein expression of Nrf2 and HO-1 in liver tissue, while reducing serum and liver MDA levels and restoring the activities of SOD and GSH-Px. Moreover, the antioxidant effect of the high-dose group (40 mg/kg) is significantly better than that of the low-dose group (20 mg/kg), further confirming that Rg1 exerts liver antioxidant protection by activating the Nrf2 pathway [44]. In addition, Korean black ginseng extracts rich in ginsenosides such as Rb1, Rg3, and Rk1 can activate the Nrf2 pathway in an Inositol-requiring enzyme 1α(IRE1α)-dependent manner in MAFLD mice induced by HFD combined with a high-fructose diet. This extract also reduces the production of ROS and nitric oxide (NO) in liver and intestinal tissues, inhibits the expression of inducible nitric oxide synthase (iNOS), and restores liver GSH and CAT activities. Its antioxidant effect is significantly weakened in IRE1α gene knockout mice, suggesting that ginsenosides may have a specific mechanism of regulating oxidative stress through the IRE1α-Nrf2 axis [45].

Nevertheless, current relevant studies have several limitations. Most experiments adopt a single dose gradient and a single modeling method; few studies explore the crosstalk between IRE1α and Nrf2 pathways under different oxidative stress intensities. In addition, nearly all published data report positive antioxidant outcomes, while negative or inconsistent experimental results are rarely published, leading to potential publication bias in existing evidence.

#### 4.1.2. Protection of Mitochondrial and ER Function

Secondly, ginsenosides can also reduce ROS production from the source by improving mitochondrial function and inhibiting endoplasmic reticulum stress (ERS)-related oxidative damage. A study by Roh et al. found that ginsenoside Mc1 can improve endoplasmic reticulum dysfunction by reducing the expression of ERS marker molecules such as Glucose-regulated protein 78 (GRP78), C/EBP homologous protein (CHOP), and Caspase12 in the palmitic acid-induced HepG2 cell model, thereby reducing ERS-mediated ROS production. At the same time, it significantly increases intracellular SOD and GSH-Px activities and reduces MDA accumulation [46]. In the diet-induced obesity (DIO) mouse MAFLD model, Mc1 intervention can restore liver mitochondrial membrane potential, reduce mitochondrial ROS release, and inhibit the mitochondrial apoptosis pathway (such as by downregulating the Bax/Bcl-2 ratio and reducing Caspase3 activation), indicating the regulatory effect of ginsenosides on these two sources of oxidative stress: mitochondria and endoplasmic reticulum [46].

Notably, existing research only verifies the protective effect of ginsenoside Mc1 on endoplasmic reticulum and mitochondria based on single-cell and animal models. There is still a lack of comparative experiments to distinguish whether the antioxidative effect originates from mitochondrial protection or endoplasmic reticulum stress inhibition alone, and the upstream regulatory mechanism remains unclear.

#### 4.1.3. Modulation of SIRT-Mediated Antioxidant Pathways

Specific ginsenosides can also enhance the ability to resist oxidative stress by regulating other antioxidant-related molecular pathways. In the mouse primary hepatocyte model induced by OA&PA (oleic acid + palmitic acid), ginsenoside Rg2 can significantly reduce ROS levels and MDA content, and increase SOD2 (mitochondria-specific SOD) expression. Its antioxidant effect is significantly weakened in SIRT1 knockout hepatocytes, indicating that Rg2 exerts anti-MAFLD effects through a SIRT1-mediated mitochondrial protection mechanism [25]. Ginsenoside Rd can activate the deacetylase activity of silent information regulator 6 (SIRT6) in HFD-induced MAFLD mice, upregulate the expression of mitochondrial respiratory chain-related proteins (such as UQCRC2), improve mitochondrial respiratory function, and reduce ROS production. Meanwhile, SIRT6 can enhance Nrf2 nuclear translocation through deacetylation modification, further amplifying the antioxidant effect. This protective effect is completely eliminated in liver-specific SIRT6 knockout mice, confirming that SIRT6 is a key target for Rd to regulate oxidative stress in MAFLD [28]. Ginsenoside Rg2 can activate the SIRT1 pathway, upregulate the expression of mitochondrial biogenesis-related genes (such as Peroxisome proliferator-activated receptor gamma coactivator-1α {PGC-1α}, Nuclear respiratory factor 1 {NRF1}, and Mitochondrial transcription factor A {TFAM}), promote mitochondrial neogenesis and functional repair, and reduce mitochondrial ROS production [25].

Current findings confirm the synergistic regulation between SIRT family proteins and Nrf2 axis, but existing studies fail to clarify the interaction difference between different SIRT subtypes. In addition, repeated experimental indicators of oxidative stress markers reduce the novelty of relevant research, and unified detection standards are absent across different laboratories.

In summary, various ginsenosides can exert antioxidant effects involving multiple signaling pathways such as Nrf2, SIRT1, and SIRT6 to effectively regulate MAFLD-related oxidative stress. Even so, the overall quality of current preclinical evidence remains limited. Most in vitro experiments adopt superphysiological high concentrations that far exceed actual human exposure levels, and rodent animal models cannot fully recapitulate long-term chronic oxidative damage occurring in human MAFLD patients. In addition, dose–effect curves vary markedly among different ginsenoside monomers, creating barriers for uniform human dose conversion and subsequent clinical translational research.

### 4.2. Anti-Inflammatory Effects

The inflammatory response constitutes the core secondary hit in the multiple-hit hypothesis of MAFLD and represents the pivotal driver that promotes disease progression from simple hepatic steatosis to MASH. As a central hub in the pathological process of MAFLD, it is not only a downstream outcome of oxidative stress and lipid metabolism disorders but also a core driving force for the further development of the disease toward liver fibrosis and even hepatocellular carcinoma.

#### 4.2.1. Inhibition of the NF-κB Signaling Pathway

The regulatory effect of ginsenosides on MAFLD inflammation is inhibiting the nuclear factor-kappa B (NF-κB) signaling pathway to block the transcription and release of pro-inflammatory factors. NF-κB is the core transcription factor regulating inflammatory responses. In the resting state, it binds to the inhibitory protein Inhibitor of kappa B alpha (IκBα) and is localized in the cytoplasm. When stimulated by lipopolysaccharide (LPS), ROS, and other stimuli, IκBα is phosphorylated and degraded, and the NF-κB p65 subunit translocates into the nucleus to initiate the expression of downstream pro-inflammatory factor genes. The study by Xiao found that in the FFA-induced HepG2 cell MAFLD model, ginsenoside Rg1 can reduce the levels of Interleukin-1β (IL-1β), Interleukin-6 (IL-6), and TNF-α in the cell supernatant in a concentration-dependent manner, and inhibit the translocation of NF-κB p65 from the cytoplasm to the nucleus, whose mechanism is closely related to downregulating the phosphorylation level of IκBα and blocking the activation of the NF-κB pathway [47].

In methionine-choline-deficient (MCD) diet-induced NASH mice, Rg1 intervention (20, 40 mg/kg) can downregulate the nuclear translocation level of NF-κB p65 in liver tissue, reduce the mRNA expression of pro-inflammatory factors, and alleviate inflammatory infiltration in liver lobules [48].

In palmitic acid-induced HepG2 cells, the panaxadiol saponin component (PDS-C) can reduce NF-κB activation mediated by lipotoxicity, and its anti-inflammatory effect is dose-dependent. In addition, the panaxadiol saponin component (PDS-C) can activate AMPK phosphorylation in HFD-induced MAFLD mice, downregulate NF-κB protein levels, and reduce the contents of IL-1β, IL-6, and TNF-α in the liver [49].

Consistent anti-inflammatory effects of ginsenosides via NF-κB inhibition are observed across cellular and animal models. However, current studies ignore the compensatory activation of other inflammatory pathways after NF-κB blockade, which may weaken the holistic anti-inflammatory effect of ginsenosides in vivo and cause inconsistent efficacy in long-term intervention.

#### 4.2.2. Suppression of NLRP3 Inflammasome Activation

Ginsenosides can reduce the maturation and secretion of pro-inflammatory factors by inhibiting NOD-like receptor pyrin domain-containing 3 (NLRP3) inflammasome activation.

In immortalized mouse Kupffer cells, the ginsenoside extract rich in Rh1 and Rg2 can reduce the expression of Cleaved-Caspase-1 and the secretion of IL-1β [50].

In MCD diet-induced MASH mice, ginsenoside Rg1 inhibits NLRP3 inflammasome activation and reduces the release of IL-1β and IL-18 by regulating the miR-375-3p/ATG2B/Phosphatase and tensin homolog (PTEN)-AKT axis and promoting the expression of autophagy-related protein ATG2B [48]. In fast food diet (FFD)-induced MAFLD mice, the ginsenoside extract rich in Rh1 and Rg2 can inhibit LPS-induced NLRP3 inflammasome activation by promoting mitophagy to clear dysfunctional mitochondria (reducing ROS production) [50]. A study by Li et al. found that ginsenoside Rg5 can significantly downregulate the protein expression of NLRP3 and Caspase-1 in the liver of MASH mice induced by HFD combined with CCl_4_, and reduce IL-1β levels, whose mechanism is related to inhibiting the Notch1 signaling pathway and reducing inflammasome activation mediated by lipid accumulation [51].

Diverse ginsenosides inhibit NLRP3 inflammasome through disparate upstream axes, but no head-to-head comparison research clarifies the most effective ginsenoside monomer. Meanwhile, combined modeling methods (HFD + CCl_4_) deviate from conventional clinical MAFLD pathological characteristics, which reduces the extrapolation value of relevant experimental data.

#### 4.2.3. Regulation of Immune Cell Polarization and Gut–Liver Axis

Ginsenosides can also improve the liver inflammatory microenvironment by regulating immune cell polarization.

In the thapsigargin (THA)-induced HepG2 cell MAFLD model, ginsenoside Rh2 can reduce the levels of pro-inflammatory factors such as IL-6, IL-1β, TNF-α, and monocyte chemoattractant protein-1 (MCP-1) in the cell supernatant. Meanwhile, the conditioned medium of HepG2 cells treated with Rh2 can inhibit the polarization of THP-1 cells (human monocyte cell line) to M1 type (reducing CD80 and CD86 expression) and promote polarization to M2 type (increasing CD163, Arg1, and MRC-1 expression), thereby alleviating liver inflammation by inhibiting hepatocyte-macrophage inflammatory crosstalk [52].

In HFD-induced MAFLD mice, ginsenoside Rk3 can significantly increase the level of intestinal short-chain fatty acids (SCFAs), regulate the composition of gut microbiota, inhibit the expression of Vascular cell adhesion molecule-1 (VCAM-1) in liver sinusoidal endothelial cells, reduce the migration and infiltration of monocytes into the liver, and further alleviate liver inflammation [53]. Korean red ginseng extract (rich in Rg3) can increase the abundance of intestinal Lactobacillus in patients with non-alcoholic silent hepatitis, reduce serum TNF-α and IL-6 levels, and improve alanine aminotransferase (ALT) and aspartate aminotransferase (AST) levels, suggesting that its anti-inflammatory effect is closely related to the regulation of the “gut–liver axis” mediated by gut microbiota [54].

It is worth noting that only one small-sample clinical study mentions the gut–liver axis regulation effect of ginseng extract, and no large-scale clinical controlled trials have verified the above outcomes. Individual gut microbiota differences among populations will greatly affect the actual anti-inflammatory efficacy of ginsenosides in human bodies.

In conclusion, ginsenosides can exert anti-inflammatory effects through multiple pathways: on the one hand, they can block the transcription of pro-inflammatory factors by inhibiting the NF-κB/MAPKs pathway; on the other hand, they can inhibit the NLRP3 inflammasome and reduce the release of IL-1β and other factors, regulate macrophage polarization to improve the inflammatory microenvironment, and also achieve anti-inflammatory effects by regulating the “gut–liver axis” through gut microbiota. Despite these consistent preclinical anti-inflammatory findings, existing studies still have notable deficiencies. Most investigations focus on short-term inflammatory alleviation without exploring long-term inflammatory memory in progressive MAFLD. Moreover, few experiments distinguish functional differences between hepatic resident macrophages and circulating macrophages, leaving the exact cellular targeting mechanisms unclear and restricting in-depth mechanistic interpretation.

From the perspective of the multiple-hit theory, oxidative stress and inflammation are interactive secondary pathological hits that form a self-amplifying vicious cycle. Current preclinical literature overwhelmingly reports positive beneficial results of ginsenosides, while conflicting experimental data and invalid outcomes are seldom reported, leading to inevitable publication bias. Additionally, all mechanistic evidence is derived from cell and rodent models, without human tissue verification. The huge gap between experimental pharmacological dose and human-safe oral dose as well as the low oral bioavailability of raw ginsenosides remain the core bottlenecks, restricting the further clinical translation of ginsenosides targeting secondary hits of MAFLD.

## 5. Ginsenosides Attenuate Terminal Cascade Damage: Dysregulated Cell Fate in MAFLD

In advanced MAFLD, persistent metabolic, oxidative, and inflammatory stress drives irreversible cell fate dysregulation, including excessive apoptosis, pyroptosis, impaired autophagy, and cellular senescence. Ginsenosides target these terminal events to limit hepatocyte loss and disease progression.

### 5.1. Inhibition of Hepatocyte Apoptosis

Excessive hepatocyte apoptosis is the key to the progression of MAFLD from simple steatosis to MASH and liver fibrosis. When affected by pathological factors such as a high-fat diet, hepatocytes can initiate apoptotic programs through mitochondrial apoptotic pathways, death receptor pathways, etc., leading to massive death of hepatocytes and inducing inflammatory responses, thereby promoting the process of liver fibrosis.

#### 5.1.1. Regulation of the Mitochondrial Apoptotic Pathway

In terms of the mitochondrial apoptotic pathway (intrinsic pathway), ginsenosides mainly exert their effects by regulating the expression of Bcl-2 family proteins and mitochondrial function. The core of this pathway is the imbalance in the ratio of anti-apoptotic proteins (Bcl-2) and pro-apoptotic proteins (Bax) of the Bcl-2 family, leading to a decrease in mitochondrial membrane potential, the release of cytochrome C, and ultimately activation of the Caspase-3 apoptotic cascade reaction.

In the FFA-induced HHL-5 hepatocyte MAFLD model, ginsenoside Rg1 can significantly downregulate the expression of the pro-apoptotic protein Bax, upregulate the level of the anti-apoptotic protein Bcl-2, reduce Caspase-3 activity, and inhibit hepatocyte apoptosis [55]. A mechanism study by Li showed that Rg1 activates the Akt/Erk1/2 pro-survival signaling pathway by downregulating the expression of sphingosine-1-phosphate lyase 1 (SGPL1), and overexpression of SGPL1 eliminates the anti-apoptotic effect of Rg1 [55].

Song et al. found that ginsenoside Rb1 can upregulate Bcl-2 expression and inhibit hepatocyte apoptosis in HFD-induced MAFLD mice by activating Peroxisome proliferator-activated receptor γ (PPARγ); the PPARγ inhibitor GW9662 can reverse this inhibitory effect [56].

Notably, current studies only focus on core apoptotic markers within the mitochondrial pathway, and few studies explore the upstream lipid stress signals that trigger mitochondrial dysfunction. Meanwhile, the efficacy difference in identical ginsenosides across different hepatocyte cell lines has not been compared systematically.

#### 5.1.2. Inhibition of the Death Receptor Pathway

In the regulation of the death receptor pathway (extrinsic pathway), ginsenosides mainly play a role by inhibiting death receptors and their downstream signals [57].

In HFD-induced MAFLD rats, ginsenoside Rg1 (20, 40 mg/kg) can downregulate the mRNA and protein expression of hepatic Fas and FasL in a dose-dependent manner, reduce the activities of Caspase-8 and Caspase-3, and decrease the number of Terminal deoxynucleotidyl transferase dUTP nick end labeling (TUNEL)-positive apoptotic cells [57]. Korean black ginseng extract (rich in Rb1, Rg3, Rk1) downregulates the expression of Fas and Tumor necrosis factor receptor 1 (TNFR1) in the intestine and liver in an IRE1α-dependent manner in MAFLD mice whose condition is induced by HFD combined with high fructose, and inhibits Caspase-8 activation to block the extrinsic apoptotic pathway [45].

Existing evidence confirms the inhibitory effect of ginsenosides on extrinsic apoptotic signals, but it remains unclear whether long-term ginsenoside intervention will cause compensatory overactivation of alternative apoptotic pathways in vivo, which may weaken long-term hepatoprotective effects.

#### 5.1.3. Modulation of Other Apoptosis-Related Pathways

In addition, ginsenosides may also exert protective effects by regulating other apoptosis-related pathways. In mouse primary hepatocytes induced by OA&PA, ginsenoside Rg2 upregulates Bcl-2 expression and inhibits Caspase-3 activation by activating SIRT1, and its anti-apoptotic effect disappears after SIRT1 knockout [58]. Lu et al. found that in HFD-induced MAFLD mice, ginsenoside Rh2 upregulates HCBP6 expression, activates the AMPK pathway, promotes fatty acid oxidation, and reduces lipotoxicity-mediated hepatocyte apoptosis, while HCBP6 knockout can weaken the anti-apoptotic effect of Rh2 [59].

Current research identifies multiple novel regulatory targets, including SIRT1 and HCBP6, but there is a lack of direct comparative experiments to clarify the dominant anti-apoptotic target of different ginsenoside monomers under consistent pathological conditions.

Multiple studies mentioned above have confirmed that ginsenosides can effectively inhibit excessive hepatocyte apoptosis in MAFLD through regulating mitochondrial apoptotic pathway, inhibiting death receptor pathway, and other apoptosis-related signals, providing key support for blocking disease progression. However, current relevant research still has prominent limitations. All anti-apoptotic mechanisms are validated in simplified in vitro hepatocyte models and rodent MAFLD models, which fail to fully recapitulate the complex internal microenvironment of progressive human MAFLD liver lesions. In addition, existing studies mostly focus on single apoptotic signaling cascades separately, lacking exploration of crosstalk between mitochondrial apoptosis and death receptor pathways. Moreover, consistent with other preclinical research fields of ginsenosides, almost no published literature reports failed anti-apoptotic intervention results, resulting in potential publication bias that weakens the overall reliability of existing evidence.

### 5.2. Uppression of Pyroptosis and Restoration of Autophagy

Pyroptosis, as a pro-inflammatory programmed cell death method, can exacerbate liver inflammation by releasing inflammatory factors; impaired autophagy leads to obstacles in clearing abnormal lipid droplets and damaged mitochondria in hepatocytes, further aggravating lipid accumulation and oxidative stress. The two together form a pathological cycle of “pyroptosis activation—impaired autophagy—metabolic disorder”, promoting the occurrence and development of MAFLD.

#### 5.2.1. Inhibition of Pyroptosis

The regulation of pyroptosis by ginsenosides is mainly achieved by inhibiting NLRP3 inflammasome activation and the expression of pyroptosis-related proteins. The NLRP3 inflammasome is the core initiating molecule of pyroptosis. In the MAFLD state, “danger signals” such as oxidative stress caused by hepatocyte lipid accumulation and ATP released by damaged mitochondria can induce NLRP3 to assemble with Apoptosis-associated speck-like protein containing a CARD (ASC) and Caspase-1 to form an inflammasome, activate Caspase-1, and promote the maturation of precursors such as IL-1β into mature pro-inflammatory factors.

Chen et al. found that in MCD diet-induced MASH mice, ginsenoside Rg1 indirectly inhibits NLRP3 inflammasome activation by regulating the miR-375-3p/ATG2B/PTEN-AKT axis and promoting the expression of autophagy-related protein ATG2B. At the same time, it reduces the levels of hepatic Caspase-1, GSDMD-N (active fragment of GSDMD), IL-1β, and IL-18, alleviating inflammatory damage mediated by pyroptosis [48]. The ginsenoside extract rich in Rh1 and Rg2 inhibits LPS-induced NLRP3 inflammasome activation and reduces pyroptosis by promoting mitophagy to clear dysfunctional mitochondria and reduce ROS production in FFD-induced MAFLD mice [50].

All included studies verify that ginsenosides suppress NLRP3-mediated pyroptosis, yet these experiments adopt different dietary MAFLD models, leading to inconsistent activation levels of baseline NLRP3 inflammasome and poor comparability among experimental data.

#### 5.2.2. Restoration of Autophagy

In terms of repairing impaired autophagy, ginsenosides mainly play a role by activating autophagy signaling pathways and restoring autophagic flux. In the MAFLD state, excessive activation of mTOR induced by high fat can inhibit autophagy initiation, and the obstacle of autophagosome-lysosome fusion caused by lipotoxicity further aggravates autophagy dysfunction.

In palmitic acid-induced HepG2 cells, ginsenoside Rb1 promotes TFEB nuclear translocation by activating transcription factor EB (TFEB), upregulates the expression of autophagy-related genes (such as Microtubule-associated protein light chain 3 {LC3}), enhances autophagic flux, accelerates lipid droplet degradation, and reduces lipid accumulation [60]. Ginsenoside Rb2 restores autophagic flux and reduces lipid droplet accumulation in HepG2 cells induced by oleic acid combined with high glucose by inducing SIRT1 and activating AMPK [35].

In the db/db mouse MAFLD model, Rb2 can upregulate the ratio of hepatic Beclin1 and LC3B-II/LC3B-I, and downregulate the expression of autophagy substrate p62, whose autophagy regulatory effect disappears after pretreatment with SIRT1 or AMPK inhibitors [35]. A study by Wang found that ginsenoside Rg1, an effective component in Tangshen Formula, can enhance the AMPK/SIRT1/autophagy pathway in MCDD-induced MAFLD mice, upregulate LC3B-II content, promote p62 degradation, restore hepatic autophagy function, and reduce lipid accumulation [33].

It can be seen that pyroptosis and impaired autophagy are important promoters of MAFLD progression. Ginsenosides can inhibit pyroptosis by suppressing the NLRP3/Caspase-1/IL-1β pathway, and restore autophagy through pathways such as TFEB and AMPK/SIRT1 to promote lipid droplet degradation, synergistically improving abnormal cell fate regulation.

Ginsenosides restore impaired autophagic flux via AMPK/SIRT1 and TFEB axes across cell and animal models, but most studies only detect static autophagy markers rather than dynamic autophagic flux, which cannot fully reflect the real autophagy regulatory status.

It can be seen that pyroptosis and impaired autophagy are important promoters of MAFLD progression. Ginsenosides can inhibit pyroptosis by suppressing the NLRP3/Caspase-1/IL-1β pathway, and restore autophagy through pathways such as TFEB and AMPK/SIRT1 to promote lipid droplet degradation, synergistically improving abnormal cell fate regulation. Nevertheless, several research gaps and translational drawbacks remain unaddressed. Most studies explore pyroptosis and autophagy as two independent pathological events, while the interactive regulatory relationship between mitophagy, pyroptosis and autophagic flux during MAFLD progression is rarely discussed. In addition, different MAFLD modeling methods, including MCD, FFD and high-glucose combined models, lead to inconsistent effective doses of identical ginsenoside monomers, and the high in vitro working concentration cannot be converted into feasible clinical oral dosage, restricting subsequent clinical application.

### 5.3. Ginsenosides Ameliorate Hepatocyte Aging in MAFLD

A study by Ogrodnik et al. found that clearing senescent hepatocytes helps reduce liver fat accumulation, while inducing hepatocyte-specific senescence exacerbates fat accumulation. This result suggests that hepatocyte senescence may be one of the key factors promoting the occurrence and development of MAFLD [61].

A study by Qi et al. found that in the d-galactose-induced fatty liver mouse model, hepatocyte senescence is manifested as an increase in the positive rate of senescence-associated β-galactosidase (SA-β-gal) staining and increased expression of senescence-related proteins p53 and p21, accompanied by a sharp increase in the phosphorylation level of forkhead box protein O1 (FOXO1), leading to decreased activity of FOXO1 in the liver. The expression of its downstream targeted antioxidant enzymes SOD and CAT is significantly downregulated; the level of lipid peroxidation marker MDA is increased, which in turn exacerbates hepatic steatosis, abnormal elevation of serum ALT and AST, and increased release of senescence-associated secretory phenotype (SASP) factors (IL-1β, IL-6, MCP-1) and lymphocyte infiltration. Ginsenoside Rg1, on the one hand, can maintain the protein level and activity of FOXO1 in the liver by inhibiting excessive phosphorylation of FOXO1, upregulate the expression of SOD and CAT to enhance antioxidant capacity, reduce MDA content, and alleviate senescence damage mediated by oxidative stress; on the other hand, it reduces hepatic steatosis and hepatocyte damage by improving FOXO1-mediated metabolic homeostasis, and inhibits SASP release and inflammatory infiltration, ultimately reversing the hepatocyte senescence-related phenotype induced by d-galactose [62].

Although there have been many studies on ginsenosides for MAFLD, the impact of senescence on the liver is complex, and there are few studies on whether anti-aging is an important pathway for ginsenosides to prevent and treat MAFLD, which still needs further exploration. Beyond insufficient research volume, the existing evidence also faces obvious limitations: current exploration is limited to a single ginsenoside Rg1 and only one aging-induced fatty liver animal model, lacking verification in conventional diet-induced MAFLD models that are more consistent with clinical human pathogenesis. Additionally, no research has clarified the crosstalk between hepatocyte senescence and the aforementioned apoptosis, pyroptosis, and autophagy disorders, failing to elaborate the status of hepatocyte senescence in the terminal cascade damage of MAFLD multiple-hit network.

Collectively, cell fate dysregulation, including apoptosis, pyroptosis, defective autophagy and hepatocyte senescence, constitutes the terminal cascade damage in MAFLD multiple-hit pathogenesis, which directly drives reversible hepatic steatosis to develop into irreversible MASH and liver fibrosis. Similar to upstream metabolic, oxidative and inflammatory research evidence, all relevant studies focusing on terminal cell fate regulation are confined to preclinical cell and animal experiments, with a complete lack of human liver tissue validation and clinical trial data. Furthermore, existing research overly focuses on the independent regulatory effect of ginsenosides on each individual cell death mode, but fails to construct an integrated regulatory network covering all terminal pathological events. The low oral bioavailability of native ginsenosides and the huge gap between preclinical effective dose and human daily safe intake are still the core obstacles limiting the clinical translation of ginsenosides targeting the terminal hits of MAFLD.

## 6. Ginsenosides Regulate the Gut–Liver Axis via Gut Microbiota

Intestinal microbiota imbalance is an important factor that persists throughout the entire onset and progression of MAFLD, and acts as a critical trigger that drives the pathological development of the disease. Under normal conditions, the gut microbiota can maintain the integrity of intestinal epithelial tight junctions, regulate the production of metabolites, and ensure the barrier effect of the intestinal barrier against harmful substances; however, in the MAFLD state, the imbalanced gut microbiota (such as an increased ratio of Firmicutes/Bacteroidetes) instead damages the intestinal barrier, causing endotoxin (LPS) to enter the blood, activating hepatic inflammatory responses through the portal vein system, and interfering with bile acid metabolism to exacerbate hepatic lipid accumulation [63]. Ginsenosides can restore the homeostasis of the gut–liver axis through the synergistic effect of “remodeling gut microbiota–repairing intestinal barrier”, and the specific mechanisms are as follows:

### 6.1. Remodeling Gut Microbiota Composition

Ginsenosides can regulate the composition of microbiota and optimize the metabolic microenvironment. In HFD-induced MAFLD mice, ginsenoside Re can significantly upregulate the abundance of beneficial bacteria *Adlercreutzia equalifaciens* and significantly reduce the Firmicutes/Bacteroidetes (F/B) ratio. At the same time, through flora-mediated regulation of bile acid metabolism, it reduces the levels of total bile acids and primary bile acids, downregulates the expression of hepatic farnesoid X receptor (FXR) and CYP7A1, and improves the enterohepatic circulation of bile acids [63]. Ginsenoside Rg5 improves microbiota imbalance in HFD-induced MAFLD mice by increasing the abundance of beneficial bacteria such as Bacteroides and Akkermansia and reducing harmful bacteria such as Olsenella. Fecal microbiota transplantation (FMT) experiments confirmed that the gut microbiota of mice treated with Rg5 can transfer its liver protective effect [27]. A study by Yang et al. found that ginsenoside Rh4 improves MAFLD induced by a Western diet combined with CCl_4_ by increasing the level of intestinal short-chain fatty acids (SCFAs, such as acetic acid and propionic acid), thus regulating microbiota diversity [64].

### 6.2. Repairing Intestinal Barrier Function

Ginsenosides can play a role by enhancing intestinal epithelial tight junctions and inhibiting intestinal leakage. The integrity of the intestinal barrier depends on the normal expression of tight junction proteins (such as Occludin and ZO-1) between intestinal epithelial cells. In the MAFLD state, inflammation induced by microbiota imbalance can degrade tight junction proteins, leading to increased intestinal permeability. In the HFD-induced MAFLD mouse model, ginsenoside Re can significantly upregulate the mRNA and protein expression of Occludin and ZO-1 in colon tissue, reduce intestinal endotoxin entering the blood, decrease serum LPS levels, and inhibit the activation of the hepatic Toll-like receptor 4 (TLR4)/NF-κB pathway to reduce the release of pro-inflammatory factors (IL-1β, TNF-α) [63]. A study by Liang et al. found that ginsenoside extract (GE) alleviates intestinal leakage and metabolic endotoxemia mediated by microbiota imbalance in HFD-induced MAFLD mice by regulating gut microbiota, and upregulates the expression of colonic tight junction proteins to repair the intestinal barrier [37]. In patients with non-alcoholic silent hepatitis, Korean red ginseng extract rich in Rg3 can improve intestinal barrier function, reduce serum endotoxin levels, and improve ALT, AST levels and fatigue scores by increasing the abundance of intestinal Lactobacillus [54].

### 6.3. Gut Microbiota-Mediated Ginsenoside Activation

Gut microbiota is also involved in the in vivo activation of ginsenosides, converting some high-content but low-activity protopanaxadiol-type ginsenosides into rare ginsenosides with higher bioavailability through stepwise deglycosylation. A study by Shin et al. found that after fermenting red ginseng with Lactobacillus, the blood concentrations of ginsenosides CY, F2, C-K, Rh2, PPD, and PPT are all higher than those seen before fermentation, indicating that most ginsenosides are more easily absorbed after fermentation [65]. A study by Dong collected the contents of rat colon to prepare intestinal microbiota solution for fermenting ginsenosides, further verifying the two main transformation pathways of “Rb1 → Rd → F2 → C-K” and “Re → Rg1 → Rh1 → PPT”. The study also found that prototype ginsenosides have extremely low oral bioavailability. After deglycosylation by intestinal flora, their metabolites show greatly improved permeability and absorption. C-K, the key final metabolite with the highest blood exposure, serves as the core bioactive component in vivo [66].

The above studies have confirmed from multiple perspectives that ginsenosides can restore the abnormal homeostasis in MAFLD by reshaping the imbalanced gut microbiota, regulating flora metabolites to optimize the gut–liver axis metabolic microenvironment, repairing the damaged intestinal barrier to reduce intestinal leakage, and realizing the transformation to high bioavailability forms with the help of gut microbiota, forming a multi-link coordinated regulatory network, which provides sufficient evidence for its use as a candidate component for gut–liver axis targeted intervention in MAFLD (Table 1). Nevertheless, current gut–liver axis-related research still has notable limitations. Most relevant experiments focus on unilateral regulation of intestinal flora or intestinal barrier separately, lacking in-depth exploration of the bidirectional crosstalk between gut microbial metabolites and hepatic pathological damage. Moreover, the included clinical evidence is limited to small-sample observational studies without rigorous randomized controlled trials, and the definite causal relationship between ginsenoside intervention, gut flora alteration and improved MAFLD symptoms cannot be fully confirmed.

## 7. Summary and Outlook

The pathological mechanism of MAFLD is characterized by the complexity of multi-link and multi-target cross-regulation. According to the widely recognized “multiple hit theory” popular at present, the metabolic imbalance formed by lipid metabolism disorder and insulin resistance constitutes the initial driving force of disease occurrence. The interaction between oxidative stress and inflammatory response accelerates pathological progression; the imbalance of hepatocyte apoptosis, pyroptosis, and autophagy further exacerbates the destruction of liver structure; and intestinal microbiota imbalance runs through the entire disease process through the “gut–liver axis”.

Collectively, current preclinical evidence demonstrates that ginsenosides act as multi-target regulators across the entire MAFLD progression cascade: targeting initial insulin resistance and lipid disorders, blocking secondary oxidative and inflammatory insults, attenuating terminal cell fate dysregulation, and restoring systemic gut–liver axis homeostasis.

Although all the evidence is obtained from preclinical in vitro cellular and in vivo animal models, the findings are highly relevant to human MAFLD. The animal and cellular models used in these studies can well recapitulate the core pathological features of human MAFLD, including insulin resistance, hepatic steatosis, oxidative stress, chronic inflammation, hepatocyte apoptosis and pyroptosis. Meanwhile, the key targets regulated by ginsenosides, such as Nrf2, AMPK, SIRT1, NF-κB and NLRP3 inflammasome, are all well-recognized therapeutic targets of human MAFLD, which provides a solid biological basis for clinical translation.

In terms of translational feasibility, ginseng is a homologous substance of medicine and food with high clinical safety. The effective doses in animal models can be converted into equivalent human doses, and long-term intervention with low-dose ginsenoside extracts (consistent with the Pharmacopoeia of the People’s Republic of China intake range) is expected to achieve preventive and adjuvant therapeutic effects in high-risk populations. In addition, the existing ginsenoside preparations and feasible formulation optimization strategies (such as improving bioavailability based on intestinal microbial transformation) further reduce the threshold of clinical translation.

However, the clinical translation still faces several obstacles: the lack of large-scale human clinical trials, the unclear synergistic effect of different ginsenoside monomers, and the difficulty in achieving high-dose effects in daily diet. Therefore, ginsenosides are more suitable as an adjuvant intervention for early prevention and metabolic management of MAFLD rather than a specific therapeutic drug. In the future, research can focus on breakthroughs in the following directions: (1) Multi-omics mechanistic exploration: use transcriptomics/metabolomics/proteomics to identify subtype-specific targets and “multi-component-multi-target” synergies; (2) Pharmacokinetic and formulation optimization: systematically study ginsenoside ADME in MAFLD models/patients; develop nano-carriers or gut-targeted formulations to improve bioavailability; (3) Promote clinical transformation research: design multi-center, large-sample, randomized controlled trials to verify efficacy and long-term safety; explore combinations with lifestyle modifications or existing drugs.

## Figures and Tables

**Table 1:** Summary of studies on ginsenosides and MAFLD.

Intervention	Model	Dosage	Key Outcome	Reference
Korean Red Ginseng (KRG)	NAFLD patients	3000 mg/d	Significantly reduced AST, ALT, γ-GT; decreased serum TNF-α; increased adiponectin	[11]
Korean Red Ginseng Extract (with Rg3)	NASH patients	2000 mg/d	Increased intestinal *Lactobacillus* abundance; reduced serum TNF-α, IL-6; improved ALT, AST; repaired intestinal barrier	[53]
Rb1	Palmitic acid-induced HepG2 cells	50 μM	Promoted TFEB nuclear translocation; upregulated LC3; enhanced autophagic flux; accelerated lipid droplet degradation	[59]
Rb1	HFD-induced MAFLD mice	10 mg/kg	Upregulated adiponectin in adipose tissue; activated hepatic AMPK phosphorylation; restored PI3K/Akt pathway; improved insulin resistance; reduced hepatic TG accumulation	[23]
Rb1	HFD-induced MAFLD mice	10 mg/kg	Upregulated Bcl-2; inhibited hepatocyte apoptosis	[55]
Rb2	db/db mice (with MAFLD)	10 mg/kg	Upregulated PPAR-α, CPT1; upregulated Beclin1, LC3B-II/LC3B-I; downregulated p62; repaired autophagy	[34]
Rb2	HFD-induced MAFLD mice	50 µmol/L	Upregulated CYP7A1; promoted cholesterol conversion to bile acids; reduced hepatic cholesterol accumulation	[35]
Rd	HFD-induced MAFLD mice	15 mg/kg	Upregulated PPAR-α-mediated fatty acid oxidation; inhibited HMGCR; activated SIRT6; promoted Nrf2 nuclear translocation; reduced ROS	[27]
Rg1	FFA-induced HepG2 cells	25, 50 μM	Reduced MDA; increased SOD activity	[42]
Rg1	FFA-induced HepG2 cells	20, 40 μg/mL	Concentration-dependently reduced IL-1β, IL-6, TNF-α; inhibited NF-κB p65 nuclear translocation	[46]
Rg1	FFA-induced HHL-5 hepatocytes	0.2, 0.4, 0.6 mM	Downregulated Bax; upregulated Bcl-2; reduced Caspase-3 activity; downregulated SGPL1; activated Akt/Erk1/2	[54]
Rg1	HFD-induced MAFLD mice	20, 40 mg/kg	Upregulated hepatic Nrf2, HO-1; reduced MDA; restored SOD, GSH-Px activity	[43]
Rg1	HFD-induced MAFLD mice	30, 60 mg/kg	Upregulated PPAR-α, CPT1, ACOX1; promoted fatty acid oxidation	[30]
Rg1	MAFLD rats	5, 10, 20 mg/kg	Increased hepatic CPT1, ACOX1 activity; improved fatty acid oxidation	[31]
Rg1	MCD diet-induced NASH mice	20, 40 mg/kg	Downregulated hepatic NF-κB p65 nuclear translocation; reduced pro-inflammatory factors; alleviated hepatic inflammatory infiltration	[46]
Rg1	MCD diet-induced MASH mice	40 μmol/L	Inhibited NLRP3 inflammasome activation; reduced IL-1β, IL-18; suppressed pyroptosis	[47]
Rg1	HFD-induced MAFLD rats	20, 40 mg/kg	Dose-dependently downregulated hepatic Fas, FasL; reduced Caspase-8, Caspase-3 activity; decreased apoptotic cells	[56]
Rg1	D-galactose-induced fatty liver mice	40 mg/kg	Inhibited excessive FOXO1 phosphorylation; upregulated SOD, CAT; reduced MDA; alleviated hepatic steatosis	[61]
Rg2	OA&PA-induced mouse primary hepatocytes	2.5, 5, 10 mg/kg	Reduced ROS, MDA; increased SOD2; activated SIRT1; promoted mitochondrial biogenesis; Upregulated Bcl-2; inhibited Caspase-3 activation; exerted anti-apoptotic effect	[57]
Rg2	HFD-induced MAFLD mice	2.5, 5, 10 mg/kg	Reduced fasting blood glucose, insulin; improved GTT, ITT; alleviated insulin resistance	[24]
Rg5	HFD-induced MAFLD mice	50, 100 mg/kg	Reduced fasting blood glucose, HOMA-IR; improved glucose tolerance; regulated gut microbiota; increased beneficial bacteria	[26]
Rg5	HFD + CCl_4_-induced MASH mice	60, 120 mg/kg	Downregulated hepatic NLRP3, Caspase-1; reduced IL-1β	[50]
Rk1	Western diet-induced MAFLD mice	Not reported	Downregulated SREBP1c, FAS, ACC; restored AMPK phosphorylation; reduced hepatic TG	[28]
Rk3	HFD-induced MAFLD mice	60, 120 mg/kg	Increased intestinal SCFAs; regulated gut microbiota; inhibited hepatic VCAM-1; reduced monocyte infiltration	[52]
C-K	Fructose-induced MAFLD mice	Not reported	Downregulated hepatic PEPCK, G6Pase; promoted GLUT4 membrane localization; enhanced glucose uptake	[25]
C-K	Type 2 diabetic OLETF rats (with MAFLD)	10, 25 mg/kg	Reduced hepatic TG, TC	[29]
Mc1	Palmitic acid-induced HepG2 cells	Not reported	Reduced ERS markers; increased SOD, GSH-Px; decreased MDA accumulation	[45]
Mc1	DIO mouse MAFLD model	10 mg/kg	Restored hepatic mitochondrial membrane potential; reduced mitochondrial ROS; downregulated Bax/Bcl-2; inhibited Caspase3	[45]
Re	HFD-induced MAFLD mice	10, 20, 40 mg/kg	Upregulated beneficial bacteria; reduced F/B ratio; regulated bile acid metabolism; downregulated FXR, CYP7A1; Upregulated colonic Occludin, ZO-1; reduced serum LPS; inhibited hepatic TLR4/NF-κB; reduced pro-inflammatory factors	[62]
Rh2	Thapsigargin-induced HepG2 cells	2.5, 5, 10 μM	Reduced IL-6, IL-1β, TNF-α, MCP-1; inhibited THP-1 polarization to M1; promoted M2 polarization	[51]
Rh2	HFD-induced MAFLD mice	25 mg/kg	Upregulated HCBP6; activated AMPK; promoted fatty acid oxidation; reduced lipotoxicity-induced apoptosis	[58]
Rh4	Western diet + CCl_4_-induced MAFLD	60, 120, 180 mg/kg	Increased intestinal SCFAs; regulated microbiota diversity; improved hepatic lipid metabolism and inflammation	[63]
PDS-C	Palmitic acid-induced HepG2 cells	Not reported	Reduced lipotoxicity-mediated NF-κB activation	[48]
PDS-C	HFD-induced MAFLD mice	Not reported	Activated AMPK phosphorylation; downregulated NF-κB; reduced hepatic IL-1β, IL-6, TNF-α	[48]
Extract (with Rh1, Rg2)	Immortalized mouse Kupffer cells	62.5 μg/mL	Reduced Cleaved-Caspase-1 expression; decreased IL-1β secretion	[49]
Extract (with Rh1, Rg2)	FFD-induced MAFLD mice	50, 150 mg/kg	Promoted mitophagy; reduced ROS; inhibited LPS-induced NLRP3 activation; suppressed pyroptosis	[49]
Extract	HFD-induced MAFLD mice	100, 200 mg/kg	Upregulated CPT-1a, ApoB100; inhibited SREBP1c; regulated gut microbiota; repaired intestinal barrier	[36]
Saponin fraction	HFD-induced rat MAFLD model	2.5, 5, 7.5, 10 mL/kg	Activated AMPK/ACC pathway; upregulated MTTP; promoted lipid transport; reduced hepatic TG	[37]

## Data Availability

No new data were created or analyzed in this study. Data sharing is not applicable to this article.

## Abbreviations

The following abbreviations are used in this manuscript:

ACC	acetyl-CoA carboxylase
ACOX1	acyl-CoA oxidase 1
ADME	Absorption, Distribution, Metabolism, Excretion
Akt	Protein kinase B
ALT	alanine aminotransferase
AMPK	Adenosine 5′-monophosphate-activated protein kinase
ApoB100	apolipoprotein B100
ARE	antioxidant response element
ASC	Apoptosis-associated speck-like protein containing a CARD
AST	aspartate aminotransferase
ATG2B	Autophagy related 2B
Bax	Bcl-2-associated X protein
Bcl-2	B-cell lymphoma-2
Beclin1	Beclin 1 autophagy protein
BMI	body mass index
CHOP	C/EBP homologous protein
Cleaved-Caspase-1	Cleaved Cysteine-aspartic acid protease-1
CPT1	carnitine palmitoyltransferase 1
CYP7A1	cholesterol 7α-hydroxylase
DIO	diet-induced obesity
ERS	Endoplasmic reticulum stress
F/B	Firmicutes/Bacteroidetes ratio
FAS	fatty acid synthase
Fas/FasL	Fas receptor/Fas ligand
FFA	Free fatty acids
FFD	fast food diet
FMT	Fecal microbiota transplantation
FOXO1	forkhead box protein O1
FXR	farnesoid X receptor
G6Pase	glucose-6-phosphatase
GLUT4	glucose transporter 4
GRP78	glucose-regulated protein 78
GSDMD-N	Gasdermin D N-terminal domain
GSH-Px	Glutathione peroxidase
GTT	glucose tolerance test
HCBP6	Hepatitis C virus core protein-binding protein 6
HFD	High-fat diet
HOMA-IR	Homeostatic model assessment-insulin resistance
IR	Insulin resistance
IRE1α	Inositol-requiring enzyme 1α
IRS-1	insulin receptor substrate-1
ITT	insulin tolerance test
IκBα	Inhibitor of kappa B alpha
Keap1	Kelch-like ECH-associated protein 1
KRG	Korean Red Ginseng
LC3	Microtubule-associated protein light chain 3
LKB1	Liver kinase B1
LPS	lipopolysaccharide
MAFLD	Metabolic dysfunction-associated fatty liver disease
MASH	Metabolic dysfunction-associated steatohepatitis
MCD	methionine-choline-deficient
MCP-1	monocyte chemoattractant protein-1
MDA	Malondialdehyde
MRC-1	Mannose receptor C-type 1
mTOR	mammalian target of rapamycin
MTTP	microsomal triglyceride transfer protein
NAFLD	non-alcoholic fatty liver disease
NASH	non-alcoholic steatohepatitis
NF-κB	Nuclear factor kappa-B
NLRP3	NOD-like receptor family pyrin domain containing 3
NRF1	Nuclear respiratory factor 1
Nrf2	Nuclear factor E2-related factor 2
PDS-C	panaxadiol saponin component
PEPCK	phosphoenolpyruvate carboxykinase
PGC-1α	Peroxisome proliferator-activated receptor gamma coactivator-1α
PI3K	phosphatidylinositol 3-kinase
PPAR-α	Peroxisome proliferator-activated receptor α
PPARγ	Peroxisome proliferator-activated receptor γ
PPD	protopanaxdiol
PPT	protopanaxatriol
PTEN	Phosphatase and tensin homolog
ROS	Reactive oxygen species
SA-β-gal	senescence-associated β-galactosidase
SASP	senescence-associated secretory phenotype
SCFAs	short-chain fatty acids
SGPL1	sphingosine-1-phosphate lyase 1
SIRT1	Silent information regulator 1
SIRT6	Silent information regulator 6
SOD	Superoxide dismutase
SOD2	mitochondria-specific superoxide dismutase 2
SREBP1c	sterol regulatory element-binding protein 1c
T2DM	type 2 diabetes mellitus
TC	total cholesterol
TFAM	Mitochondrial transcription factor A
TFEB	transcription factor EB
TG	Triglycerides
THA	thapsigargin
THP-1	THP-1 human monocytic cell line
TLR4	Toll-like receptor 4
TNF-α	Tumor necrosis factor-α
TNFR1	Tumor necrosis factor receptor 1
TUNEL	Terminal deoxynucleotidyl transferase dUTP nick end labeling
VCAM-1	Vascular cell adhesion molecule-1
VLDL	very low-density lipoprotein
γ-GT	gamma-glutamyl transferase
